# Cognitive Remediation Virtual Reality Tool a Recovery-Oriented Project for People with Bipolar Disorder: Protocol of a Feasibility Randomized Clinical Trial

**DOI:** 10.2174/17450179-v18-e2208220

**Published:** 2022-10-06

**Authors:** Alessandra Perra, Valerio De Lorenzo, Rosanna Zaccheddu, Aurora Locci, Federica Piludu, Antonio Preti, Lorenzo Di Natale, Alessia Galetti, Antonio Egidio Nardi, Giulia Cossu, Federica Sancassiani, Simone Barbato, Ottavio Cesaretti, Peter Konstantin Kurotshka, Mauro G. Carta

**Affiliations:** 1 International Ph.D. in Innovation Sciences and Technologies, University of Cagliari, Cagliari, Italy; 2 PRoMIND Services for Mental Health, Rome, Italy; 3 Department of Medical Sciences and Public Health, University of Cagliari, Cagliari, Italy; 4 Department of Neuroscience, University of Turin, Turin, Italy; 5 IDEGO Digital Psychology Society, Rome, Italy; 6 Federal University of Rio de Janeiro, Rio de Janeiro, Brazil; 7 Department of General Practice, University Hospital Wuerzburg, Wuerzburg, Germany

**Keywords:** Virtual reality, Cognitive remediation, Mental health, Recovery, Psychiatric rehabilitation, Trial

## Abstract

**Introduction:**

Cognitive deficits are considered a fundamental component of bipolar disorder due to the fact that they negatively impact personal/social functioning. Cognitive remediation interventions are effective in the treatment of various psychosocial disorders, including bipolar disorder. The use of Virtual reality as a rehabilitation tool has produced scientific evidence in recent years, especially in cardiovascular, neurological, and musculoskeletal rehabilitation. This study aims at evaluating the feasibility of a Cognitive Remediation Virtual Reality Program (CEREBRUM) for people with bipolar disorder in psychiatric rehabilitation.

**Material and Methods:**

Feasibility randomized controlled cross-over clinical study; we randomized 50 people from the Consultation and Psychosomatic Psychiatry Center of the University Hospital of Cagliari (San Giovanni di Dio Civil Hospital) with a diagnosis of bipolar disorder. We propose a cognitive remediation program in virtual reality (CEREBRUM), 3 months with 2 weekly sessions, for the experimental group and a usual care program for the control group (psychiatric visit and/or psychotherapy).

**Results:**

The results of the trial will be published in international peer-reviewed journals and will be disseminated at international meetings and congress.

**Discussion:**

This RCT aims, with regards to its feasibility and design, to provide information about a confirmatory trial that evaluates the effectiveness of a Virtual Reality Cognitive Remediation program in psychiatric rehabilitation for the treatment of cognitive dysfunction in people with bipolar disorder.

**Conclusion:**

The results that we analyzed at the end of the RCT will have an impact on psychiatric rehabilitation research with a focus on improving the application of technologies for mental health.

Trial registration: ClinicalTrialsgov NCT05070065, registered on September 2021.

## 1 INTRODUCTION

Bipolar disorder is a range of chronic diseases characterized by recurrent episodes of mania/hypomania, depression, and euthymia (mood dysregulation); dysregulation in sleep/wake rhythm, and medical and psychiatric comorbidities [1]. Community surveys found that the lifetime prevalence of Bipolar disorder ranges between 1% and 2.4%, although the methodology of published studies may underestimate its prevalence [2]. The disease is highly disabling due to its early onset, severity and chronicity: it is considered one of the leading causes of disabilities in the world [3], accounting for 7.0% of all DALY’s due to mental and substance use disorders [4-6]. Alongside effective symptoms, cognitive impairment is believed to be a core component of bipolar disorder [7], and in the long-term bipolar disorder was found to be associated with a high risk of dementia [8, 9]. Overall, approximately 40% to 60% of people that experienced one or more episodes of bipolar disorder have neurocognitive impairment [10]. Neurocognitive dysfunctions may be found in premorbid stages, before the disease onset [11], in the early course of the illness [12], and during the euthymia phase [13]. Poor cognitive performance impacts negatively on occupational and social functioning [14], increases the hospital admissions, affects direct/indirect healthcare related costs [15], hinders the benefits of psychotherapy [16], and represents a barrier to achieving adequate social and occupational functioning and a good quality of life [17]. The affected domains included attention, processing speed verbal learning/memory, memory, and executive functions including cognitive flexibility, inhibitory control, and working memory [18, 19]. Despite the growing attention paid to identifying and treating cognitive impairment in bipolar disorder, evidence is still lacking on effective interventions aiming to minimize cognitive symptoms in these patients [20]. Lithium was deemed to have an indirect positive effect on cognition in bipolar disorder, but other drugs used in the treatment of bipolar disorder entail cognitive side-effects related with extrapyramidal, sedative, anticholinergic, and blunting mechanisms [21]. The term Cognitive Remediation (CR) implies interventions based on behavioral training that aims to improve cognitive processes (memory, attention, executive functions, social cognition, and metacognition), which have to be exerted long enough to guarantee the persistence of the results and their generalization [22]. Different approaches were used, ranging from paper-and-pencil to computerized-based rehabilitation program to mixed interventions [23, 24]. Initial evidence suggests that CR and physical activity may exert protective effects for the prevention of cognitive decline [25], and CR has been found to be effective in the treatment of various psychosocial disorders (including bipolar disorder) to improve the cognitive and social-occupational domains, although the evidence base is still inconclusive [20, 26, 27]. Recently, Virtual reality (VR) emerged as a promising rehabilitation tool, with a growing number of studies suggesting that VR may facilitate learning and/or enhance different skills, thanks to the ability to make learning experiences real and ecological compared with traditional intervention techniques [28-30]. Current research on the clinical use of VR software has led to positive results, especially in cardiovascular, neurological, and musculoskeletal rehabilitation [31. 32]. In mental health, the evidence is limited to rehabilitation of social cognition in people with schizophrenia, and the psychotherapy of anxiety disorders and phobias, it is increasing the focus on the use of VR for CR interventions especially for schizophrenia and mild cognitive impairment [33]. To date, there are no studies in which VR was used as a CR intervention to improve the cognitive processes related with the personal and social functioning of people diagnosed with bipolar disorder. Given the ability of VR to facilitate and generalize the learning of the trained skill and its capacity to favor engagement in the task [30] and that CR may exert protective effects for the prevention of cognitive decline, therefore, it is important to promote randomized controlled clinical trials that evaluate the effectiveness of VR-implemented CR interventions. CEREBRUM is one of the first VR-implemented CR tools in the field of psychiatric rehabilitation in Europe, conceived, and designed by “PRoMIND - Services for mental health SRLS” (Rome) in association with “IDEGO - Virtual Psychology” (Rome).

### 1.1 Objectives

#### 1.1.1 Primary Objectives

Feasibility assessment of a confirmatory trial to evaluate the effectiveness of a VR-implemented cognitive remediation tool (“CEREBRUM”) for the treatment of cognitive deficits in people with bipolar disorder.

#### 1.1.2 Secondary Objectives

Preliminary evaluation of the intervention’s safety, participant satisfaction, and clinical effectiveness.

## 2 MATERIALS AND METHODS

### 2.1 Study Design

This is a randomized, controlled cross-over clinical feasibility study. This study follows the CONSORT flow diagram extension for feasibility study [34]. The protocol has been written according to the Standard Protocol Items (SPIRIT checklist) [35].

### 2.2 Participant Identification

The participants with psycho-social disabilities were recruited at the Consultation and Psychosomatic Psychiatry Center of the University Hospital of Cagliari (San Giovanni di Dio Civil Hospital).

*Inclusion Criteria:* age from 18 to 75; diagnosis of bipolar disorder according to DSM-IV [36]; both sexes; users who sign the informed consent; users under protection for which the informed consent is signed by the support legal administration.

*Exclusion Criteria:* The non-satisfaction with the inclusion criteria; *the presence of maniac/depressive phases;* the diagnosis of epilepsy or serious eye diseases, due to the risk associated with the excessive stimulation of virtual reality.

### 2.3 Randomization

Eligible participants were randomized into two groups, using a computer-generated randomization list. The biometrician responsible for the randomization process was blinded to the identities of participants. The experimental group (A) receives the CR intervention with virtual reality (CEREBRUM) lasting 3 months (two weekly meetings) and the control group (B) receives treatment as usual. When group A receives the “CEREBRUM” intervention, group B receives treatment, as usual, 1 month after the end of the intervention for group A (washout period) group B receives the “CEREBRUM intervention becoming experimental group and group A the routine intervention (Table **1**).

### 2.4 Blinding

The nature of the intervention does not permit the blinding of the participants or the mental health workers on the project.

### 2.5 Intervention

CEREBRUM is Immersive Virtual Reality software, developed by professionals and experts operating in the field of cognitive rehabilitation (psychiatric rehabilitation technicians and psychologists). It is compatible with the Oculus Go virtual reality viewer, a device with CE obligation. The CEREBRUM App allows the user to immerse themselves in experiential situations that simulate everyday reality, useful for working on users' resources, and difficulties. The user wearing the viewer sees a virtual environment that can be explored at 360 °. The user does not interact with the virtual environment but explores the scene and answers the rehabilitator's questions. It reinforces the improvements obtained from an intervention based on the Cognitive Remediation approach, also allowing direct monitoring by the health worker.

The CEREBRUM App consists of 52 exercises of varying difficulty: 22 belonging to the Memory, and Learning Module, 10 to the Cognitive Estimates Module and 20 to the Attention and Working Memory Module.

The different degrees of difficulty are designed to adapt to the user's functional diagnosis. The clinician must adapt the difficulty level to the residual abilities of the user so that the exercises are neither too easy nor too complex.

The intervention involves 24 sessions of 45minutes, 2 sessions per week for 3months. Each session was structured as follows: Reception, psychoeducation and orientation to the tool; Exercise psychoeducation; Psychoeducation to the function trained by exercise; Generalization phase, in which the function and its importance in the context of life are explained to the person (a bio-psycho-socio-cultural approach based on cognition); Carrying out the exercise in VR with positive and corrective feedback; Post-exercise comment/return; Second exercise with the same method mentioned above; The exposure within the Virtual Reality must be a maximum of 15-20 minutes; Final return; Homework to leave, intended as practical suggestions that the patient must try to carry out during his day.

In general: 1 Attention and Working Memory exercise plus 1 Memory/Learning exercise or 1 Cognitive Estimation exercise. In some sessions, depending on the user, the session, and the operator's assessment, you can also do a third exercise belonging to any area.

### 2.6 Control

The control group receives treatment, as usual, consisting of a psychiatric consultation with or without psychotherapy.

### 2.7 Outcomes

The Primary outcome is defined as the proportion of patients recruited among those considered eligible. Coprimary outcome is the proportion of patients completing the trial intervention among those included.

Secondary outcomes:

- Intervention’s safety: number of adverse events and severe adverse events
- Patients satisfaction
- Clinical Effectiveness in improving the cognitive process, personal, and social functioning, levels of perceived anxiety, quality of life, emotional awareness, and psychopathological symptoms. An intervention protocol person-centered and recovery-oriented [37], is defined as a process of change through which the individual improves their health and well-being (lives in a “self-directed” way and is committed to living to the best of their potential), whether it may lead to a global improvement.

### 2.8 Data collection

The data collection was made with a personal data sheet; consecutively the patients were enrolled at the Consultation and Psychosomatic Psychiatry Center of the University Hospital of Cagliari (San Giovanni di Dio Civil Hospital). The presence of possible side effects and satisfaction with the intervention was assessed through a self-report questionnaire, for the others, secondary outcome indicators were used, and a standardized evaluation tool validated in Italian and used in psycho-social research (in the case of a learning effect, different versions for retests were used). Participants were assessed before the treatment, after the end of the intervention, and after 6 and 12 months after the end of the intervention.

*For the cognitive evaluation was used:* Matrix test [38]; Rey Figure Test [39]; Rey's Words Test, in the two versions [40, 41]; Digital Symbol Substitution Test, in the two versions [42, 43]; Trail Making Test, in the two versions [44]; Normal and Inverse Digit Span, in the two versions [45, 46]; Stroop Test [47]; Frontal Assessment Battery - FAB [48]; Phonological and Semantic Verbal Fluency Test, in the two versions [41, 49]; Test of Cognitive Estimates (CET), in the two versions [50, 51]; Test of the Tale [52, 40].

*For the general evaluation was used:* SF-12, Short Form Health Survey with 12 items [53], a self-administering scale, which investigates the following dimensions of well-being: vitality, physical function, physical pain, perception of general health, mental health, physical and emotional, work functioning, and social role. TAS-20 Toronto Alexithymia Scale [54] self-administering scale, evaluates the level of emotional awareness. Self-Rating Scale (SAS) self-administered scale [55] evaluates perceived anxiety levels regardless of diagnosis. The Patient Health Questionnaire - PHQ-9, self-administering scale [56], evaluates depressive symptoms. Health of The Nation Outcome Scale - HoNOS [57] evaluates personal and social functioning and clinical performance. Biological Rhythms Interview of Assessment in Neuropsychiatry - BRIAN validated in Italian version [58], an interview consisting of 18 items that investigates 4 main areas related with the dysregulation of circadian rhythms (sleep, activity, social rhythms, and nutrition).

### 2.9 Sample Variables

Gender and age; Marital status; Educational qualification; Previous and current employment status; Past and current organic physical pathologies; Previous and current mental health diagnosis; Drugs in use.

### 2.10 Data Analysis

The outcome of the trial was analyzed through multivariate analysis of variance (MANOVA). Through this procedure, the sample means of the dependent variables (scores in the performance tests) were compared in sub-samples divided by the two independent variables [time and group (intervention and non-intervention)]. For variables not on an interval scale, the analysis of variance for nominal data by De Castellan was used with a similar methodology.

### 2.11 Sample Size Considerations

To date there is still poor evidence in this field of research for establishing an effective methodology in terms of sample size, and in this sense, the aim of this study is to verify the feasibility [20, 59]. We randomized 50 participants in order to be able to assess the feasibility outcomes: recruitment and retention rates Fig. (**1**).

### 2.12 Trial Status

The study is registered on ClinicalTrials.gov (NCT05070065). The Regional Ethical Committee has approved the study (PG/2020/21681). The study started before the pandemic situation, after the lockdown, we had to interrupt the study. It was repeated and definitely started again in August 2021. Actually, the participants are receiving the VR-CR program.

## 3 RESULTS

The results of the trial will be published in international peer-reviewed journals and will be disseminated at international meetings and congress.

## 4 DISCUSSION

The primary aim of this trial is to study the feasibility (recruitment and retention rates to inform sample size calculations to confirmatory trials) of a VR-implemented CR tool (CEREBRUM), secondary to study cognitive and psychosocial variables. The hypothesis is that through a cognitive remediation virtual reality program, and using ecological instrument, it is possible to improve cognitive abilities and also the other social and personal outcomes. Their results were discussed with all the authors.

### 4.1 Risk and Benefits

The use of immersive VR could have different side effects such as dizziness, nausea, headache, eye fatigue, reduced limb control, reduced postural control, reduced sense of presence, and development of inadequate responses to the real world. As a matter of fact, important side effects are not expected, as the VR tool has already been used in people with psychosocial disabilities, albeit for other purposes, without substantial side effects [60-64].

## CONCLUSION

Mental health is a fundamental resource that allows people to achieve daily goals in life and exercise the role of a citizen of a community. Bipolar disorder is recurrent condition that severely impacts the lives of people living with the experience; it is considered one of the leading causes of disabilities in the world [3]. To date, there is a lack of evidence for the treatment of cognitive deficits in BD even if cognitive deficits are a core component of personal and social functioning. The principle neuropsychological deficits are in attentional capacities, executive functions, and episodic/verbal memory [9]. The application of innovation science is increasing in the mental health field and the use of technologies can have a positive impact on psychiatric rehabilitation. The crossover study is a clinical study in which each group consecutively receives the treatments subjected to the study. This guarantees as an advantage a low variance given that the treatment and control correspond to the same participant (allows precise comparisons between multiple treatments) and it also guarantees the provision of the clinical intervention to all participants. The results of this study might have an impact on future studies, in particular, in the field of psychosocial intervention for the treatment of cognitive dysfunction in bipolar disorder and also in the technological innovation field for mental health rehabilitation.

## Figures and Tables

**Fig. (1):**
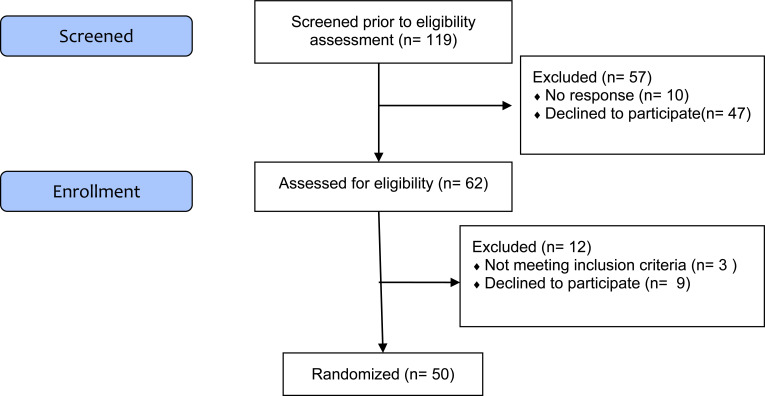
CONSORT flow diagram extension for a feasibility study (stopped at the current trial status).

**Table 1:** Gantt diagram of the study design.

**Month**	**1**	**2**	**3**	**4**	**5**	**6**	**7**	**8**	**9**	**10**	**After 6 Months from the Treatment**		**After 12 Months from the Treatment**	
**Recruitment**														
**Eligibility screen**														
**Informed consent**														
**Randomization**														
**Intervention**				**Group A**		**Group B**				
**Assessment**														
**Follow-up**														

## Data Availability

The authors confirm that the data supporting the findings of this study are available within the manuscript.

## LIST OF ABBREVIATIONS

CEREBRUM: = Cognitive Remediation Virtual Reality Program
VR: = Virtual Reality
SAS: = Self-Rating Scale
